# Outcomes of laparoscopic repair in complicated groin hernia: A single institutional based cohort study in Nepal

**DOI:** 10.1016/j.amsu.2022.104466

**Published:** 2022-08-28

**Authors:** Bhawani Khanal, Sunit Agrawal, SashiShekhar Adhikari, Robal Lacoul, Abhijeet Kumar, Rakesh Kumar Gupta

**Affiliations:** Department of General Surgery and MIS Unit, B P Koirala Institute of Health Sciences, Dharan, Nepal

**Keywords:** Complicated groin hernia, Totally extra-peritoneal repair, *Trans*-abdominal preperitoneal repair, EHS, European Hernia Society, TAPP, Trans-abdominal Pre-peritoneal, TEP, Totally Extra-peritoneal

## Abstract

**Introduction:**

Laparoscopic hernia repairs have comparable recurrence rate with less persisting pain and numbness and quicker return to usual activities as compared to open mesh repair. The excellent outcomes of minimally invasive surgery encourage us to extend the laparoscopic technique to complicated groin hernia.

**Method:**

A total of 22 patients with complicated groin hernia who presented to our institute from September 2017 to September 2018 were included in this prospective cohort study. Inclusion criteria were patients with age greater than 18 years and clinically diagnosed as complicated groin hernia. Patients with peritonitis, those with previous abdominal surgery and unfit for general anesthesia were excluded from our study.

**Results:**

The most common age group was 51–60 (31.8%) years.17 cases were repaired with totally laparoscopic approach (12 TEP, 5 TAPP). Laparoscopic repair with additional procedure was needed in 3 cases and 2 cases were converted to open for completion of the procedure. The mean operating time was 154.8 ± 51.6 (range: 90 to 230) minutes. The average length of hospital stay was 3.8 ± 3 (range: 1 to 12) days. Bleeding from the inferior epigastric and testicular vessels were the major intra-operative complication (11.8%). Seroma and surgical site infection were seen in 2 (11.8%) patients.

**Conclusion:**

Laparoscopic approach in cases of complicated groin hernia can achieve desirable patient outcomes without major complications, provided good patient selection and expertise. The evidence for laparoscopic repair as the choice of procedure in complicated groin hernia can be established from further comparative studies.

## Highlights

• Laparoscopic repair in complicated hernia has been attempted more frequently due to availability of expertise and detailed knowledge of anatomy.
• Successful reduction of hernia contents in laparoscopic repair is possible with minimal complications.
• Complications in laparoscopic repair in complicated hernia (intraoperative/postoperative) are comparable to that of laparoscopic repair of uncomplicated hernia or open repair of complicated hernia.
• Mesh can be used in laparoscopic repair of uncomplicated hernia.

## 1 Introduction

Trans-abdominal preperitoneal [TAPP] and totally extra-peritoneal [TEP] approaches are the two most accepted techniques for laparoscopic repair of groin hernias. Complicated groin hernia [irreducible, obstructed, strangulated and incarcerated] is a common surgical emergency [1].Laparoscopic operations offer the advantages of rapid postoperative recovery and reduced hospital stay [2].Laparoscopic hernia repair in the elective setting has been shown to have comparable recurrence rate with less persisting pain and numbness as well as quicker return to usual activities when compared with open mesh repair [3].However, complicated groin hernia has traditionally been seen as a relative contraindication for laparoscopy approaches due to technical difficulties with regards to reduction of the incarcerated hernia contents, increased risk for iatrogenic injuries and increased morbidity.

With the experience obtained in elective laparoscopic groin hernias repair, there is increasing confidence in both the surgical technique and understanding of the pre-peritoneal anatomy. This together with the experience obtained from laparoscopic management for other surgical emergencies has led surgeons to perform laparoscopic treatment for emergency groin hernias [4].In 2013, the European Association for Endoscopic Surgery concluded that laparoscopy can be applied for treatment of incarcerated inguinal hernias [5].Moreover, the laparoscopic approach allows thorough internal abdomen exploration to evaluate organ viability and also provides sufficient time to make decisions regarding bowel resection. Another advantage of laparoscopy is that it facilitates diagnosis and treatment of contralateral hernias [6].The excellent outcomes in terms of postoperative quality of life, measured by postoperative pain control, rapid recovery of daily activities with return to work and the grade of patients' satisfaction encourage to extend the laparoscopic technique to incarcerated inguinal hernia in urgency [7]. In this study, we tried to evaluate the outcomes of laparoscopic repair in complicated groin hernia.

## 2 Method

A total of 22 patients who presented to the casualty of a tertiary care center in Eastern Nepal from September 2017 to September 2018 with diagnosis of complicated groin hernia were included in this prospective cohort study. Complicated groin hernia includes irreducible, obstructed, strangulated and incarcerated hernia. The patient's characteristics, operative details, duration of hospital stay, incidence of complications, mortality and recurrence were observed. Patients with age greater than 18 years with diagnosis of complicated groin hernia were included in our study. Patients with features of peritonitis, history of previous abdominal surgery and those unfit for general anesthesia were excluded from our study. All consecutive patients meeting eligibility criteria were recruited in the study after written consent was obtained. No incentives of any nature were provided to the participants. Patients were evaluated in the follow-up clinic at 1, 4, 12 weeks and 6 months after the intervention done for complicated groin hernia. Further, telephone contacts were made to the patient to assess for any subjective symptoms at the end of two years. All data were prospectively entered into a structured proforma. The study was performed in accordance with the principles of the “Declaration of Helsinki” and after approval by the Institutional Review Committee [Code No: IRC/1217/018] of our institute. The study is registered at ClinicalTrials.gov [NCT05107986]. This study has been reported in line with the STROCSS 2021 criteria [8].

### 2.1 Surgical technique

All patients were kept Nil per Oral with intravenous fluids, nasogastric tube placement, parenteral antibiotic (Injection Ceftriaxone). All surgeries were performed under general anesthesia by a single surgeon. The type of laparoscopic approach (TEP or TAPP) to be performed was decided by the operating surgeon and was performed using a standard 3 port technique.

The reduction of content was done by gentle pulling from inside with non traumatic instruments and external manual compression. If the content was not reduced with this maneuver, the hernia ring was cut in a ventro-lateral direction in indirect inguinal hernia and in ventro-medial direction for direct hernia. After content reduction, using standard technique of preperitoneal dissection, space of Retzius, space of Bogros, superior and inferior space was made, with aim to achieve the critical view (CV) of the myopectineal orifice (MPO). The sac was dissected from cord structure and peritoneum was dissected sufficiently to parietalize the cord's elements. 12 × 15 macroporous polypropylene mesh was introduced through telescopic port and spread to cover the myopectineal orifice. It was followed by control release of pneumopreperitoneum. Recheck laparoscopy for secondary bowel assessment was carried out after completion of laparoscopic hernia repair. If bowel resection was required, involved bowel was brought out through transverse abdominal incision and bowel was then returned to the abdominal after completion of the procedure.

Operative time and intra-operative complications such as vascular, nerve or vas deferens injury, peritoneal breach and pneumoperitoneum were noted. After the surgery, a standard analgesic regimen was administered (IV Paracetamol 1 g 6-8hourly for 24 hours). Postoperative pain was evaluated both qualitatively and quantitatively; quantitatively need of any extra analgesic [intravenous Tramadol] was noted followed by oral diclofenac sodium 50 mg on demand for pain relief and qualitatively visual analogue scale (VAS) pain scoring system was used to monitor the postoperative pain on 12, 24 and 48 hours.

Feeding was resumed after full regain of consciousness and passage of flatus. Postoperatively hematoma, seroma, subcutaneous emphysema, wound infection and early recurrence were noted. Patients were assessed by independent surgeons for discharge considering diet, ambulation and requirement of oral analgesics if any. All the patients were encouraged to resume normal activity after surgery including work and sports, when they felt able to do so. The pain and other symptoms were recorded and clinical examinations were conducted to look for any recurrence at one, four and twelve weeks. Those with problems were followed up for a longer period as far as possible. Patients unable to attend the follow-up clinic were assessed by telephonic conversation.

### 2.2 Statistical measures

The continuous outcomes were expressed as Mean (Standard Deviation) or Median (Interquartile range); categorical outcomes were expressed as Frequency (Proportion). Shapiro Wilk test was used to assess normality of the continuous outcomes.

## 3 Results

**Fig. 1:**
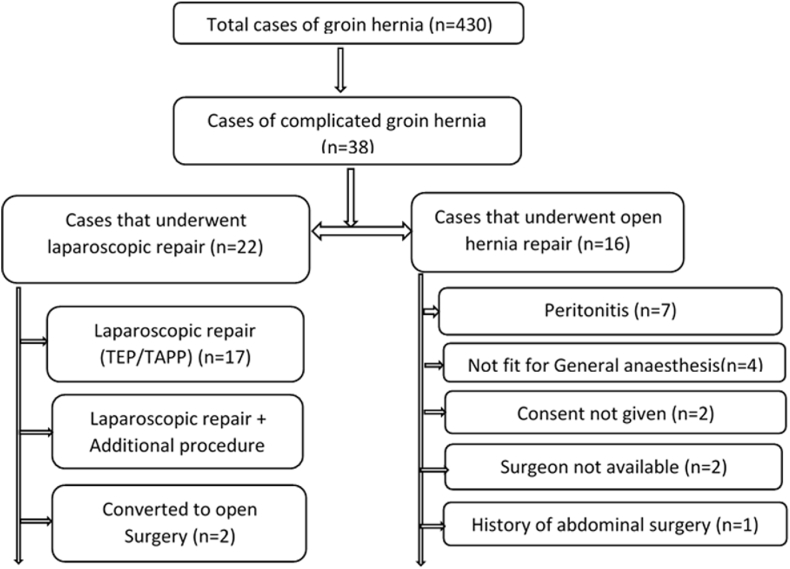
Consort flow chart of our study.

During study period, 430 patients presented with groin hernia in our institute. Among them 38 patients [8.8%] presented with complicated groin hernia, out of which only 22 patients underwent laparoscopic hernia repair and were included in our study. Remaining 16 cases were excluded because among them 7 cases presented with perforation peritonitis, 4 cases were not fit for general anesthesia, 2 cases didn't give consent for laparoscopy, 1 case had history of major abdominal surgery and 2 cases had to undergo open repair because the operating surgeon was not available (Fig. 1).

The mean age of the patients in our study was 51.4 ± 15.7ranging from 22 years to 77 years. Majority of the patients were male 19 (86.4%). Most of the patients had presented with hernia for less than a year with a median duration of 11.5 months (IQR: 2.7–24 months).More than half (54.5%) had right sided presentation. Pain at the hernia site was present in all the patients while irreducibility of the hernia contents was present in 21 patients (95.5%). Most of the patients (40.9%) had presented with obstructed groin hernia. Three patients (13.6%) had features of obstruction with signs of localized peritonitis which were attempted for laparoscopic repair after informed consent with risk of conversion. There was presence of gangrenous bowel (1) and gangrenous omentum (2) in those cases which required additional procedure (Table .1).

**Table 1:** Clinico demographic profile.

Age	
*41-50 years*	9 [41%]
*51-60 years*	7 [31.8%]
*61-70 years*	6 [27.2%]
Sex	
*Male*	19 [86.4%]
*Female*	3 [13.6%]
Duration of hernia	
*1-5 years*	8 [31.8%]
6 months *– 1 year*	6 [36.4%]
*1-*6 months	8 [31.8%]
Distribution of hernia	
*Right*	12 [54.5%]
*Left*	8 [36.4%]
*Bilateral*	2 [9.1%]
Symptoms	
*Pain*	22 [100%]
*Irreducibility*	21 [95.5%]
*Vomiting*	15 [68.2%]
*Not passing stool and flatus*	13 [59.1%]
Clinical classification of hernia	
*Obstructed*	9 [40.9%]
*Irreducible*	8 [36.4%]
*Strangulated*	3 [13.6%]
*Incarcerated*	2 [9.1%]

Majority (n = 18) of the hernia were indirect inguinal (Table .2). Most of the cases were operated within 24 hours of presentation with an average duration of 16.7 ± 6.6 (range: 6–28) hours.

**Table 2:** Distribution of hernia according to EHS classification.

Type of hernia	EHS 1	EHS 2	EHS 3	Total
Direct hernia Medial [M]	0	0	1 [4.5%]	1 [4.5%]
Indirect hernia Lateral [L]	1 [4.5%]	10 [45.5%]	7 [31.8%]	18 [81.8%]
Femoral hernia Femoral [F]	0	3 [13.6%]	0	3 [13.6%]

Out of the 22 patients, 13 patients underwent TEP (59.1%) among which one case had to be converted to open for doubtful viability of the testis. TAPP was done in 9 patients (40.9%) among which 4 had to undergo additional procedure. Among these 4 cases, 2 cases (9.1%) needed only additional trans-abdominal incision for primary repair of iatrogenic enterotomy (1) and resection of gangrenous omentum (1), while other 2 cases (9.1%) needed inguino-scrotal exploration as there was presence of gangrenous ileal segment (1) and huge sac with sliding sigmoid colon and gangrenous omentum (1).(Table .3).

**Table 3:** Type of surgery performed.

Surgery	TEP [n [%]]	TAPP [n [%]]
Totally laparoscopic repair	12 [54.6%]	5 [22.7%]
Laparoscopic repair + Additional procedure	1[4.5%]	2[9.1%]
Conversion to open	0	2 [9.1%]

Most of the hernias (36.4%) were reduced after external manipulation post creating pneumoperitoneum. Need of intraoperative ring extension was required in 9.1% hernias (Table .4).

**Table 4:** Methods of reduction of hernia contents.

Method	n [%]
Pre-operative external manipulation	5 [22.7%]
Post general anesthesia	5 [22.7%]
External manipulationpostpneumoperitoneum	8 [36.4%]
Intraoperative ring extension	2 [9.1%]
Conversion to open	2 [9.1%]

Small bowel was present as content of the hernia sac in 11 patients (50%) followed by omentum which was present in 8 patients (36.4%). Two patients (9.1%) had empty sac as the hernia sac was reduced pre-operatively.

The placement of polypropylene standard weight mesh was done in all the patients except one who underwent primary suture repair after resection-anastomosis of gangrenous ileal segment.

The mean operating time was 154.8 ± 51.5(90–230) minutes. The operation time was longer in those cases which needed additional procedure (216 ± 11.4 vs 136.8 ± 44.1) minutes.

Bleeding from inferior epigastric vessels and testicular vessels occurred in a single patient (5.9%) each which was dealt using electrocautery/harmonic sealing device. Peritoneal breach occurred in 2 patients (11.8%) who underwent TEP repair and the defect was repaired using titanium clips and use of endoloop made of polydiaxone (PDS) no 1 suture. Another single case had bowel injury for which additional hybrid technique was applied and primary repair of bowel perforation was done (Table .5).

**Table 5:** Complications [intraoperative and postoperative].

Intraoperative complications	
*Bleeding [epigastric/testicular vessels]*	2 [11.8%]
*Bowel injury*	1 [5.9%]
*Peritoneal tear*	2 [11.8%]
Postoperative complications	
*Seroma*	2 [11.8%]
*Urinary retention*	3 [17.7%]
*Wound infection*	2 [11.8%]

Seroma was present in 2 patients (11.8%) which got resolved after few days. There were 2(11.8%) superficial surgical site infections occurring in patients who underwent additional procedure and were managed with daily dressing and antibiotics. Urinary retention was present in 3 (17.7%) patients who required Foley's catheterization postoperatively. However, urinary retention couldn't be assessed in 10 patients as they were catheterized pre-operatively(Table .5).

Eleven patients (50%) needed additional analgesic in immediate postoperative period with a mean VAS of 6 at 12 hours. Average number of additional analgesic dose required was 1.7(∼2). The pain significantly reduced after 24 hours of surgery with a mean VAS score of 3.5 at 48 hours.

### 3.1 Discharge and follow-up

The average length of hospital stay in our study was 3.78 ± 3(range: 1 to 12) days. The average length of hospital stay was longer in patients who needed additional procedures (9.2 vs 3.8 days).The average number of days after which they resumed their normal daily activities was 7.5 ± 2.9 (range: 4 to 13) days with patients who needed conversion taking more days (12 vs 7.5). The mean follow-up duration at the clinics was 5.4 ± 2.2 (range: 3 to 10) months. All the patients were followed up for a minimum duration of 6 months. A single case was lost to follow up after 12 weeks. Patients were followed-up via telephonic conversation to assess about the persistence of pain and recurrence of hernia. Only 2 patients (9.1%) had moderate pain requiring occasional intake of oral analgesics at the end of first year. However, it was not severe enough to hamper their daily normal activities. The patients assessed for subjective symptoms, through telephonic conversation, at the end of second year reported no complaints. The two participants who had reported pain at the end of first year were asymptomatic a year later.

## 4 Discussion

Although traditional open approach has always been the procedure of choice in cases of complicated groin hernia, this approach has shown higher recurrence rate along with possible contamination of the mesh and lack of time for proper evaluation of ischemic bowel in cases of complicated groin hernias. Meanwhile, combined laparoscopic approach enables easy reduction of the contents with enough time for assessment of viability of the bowel and offers minimal risk of mesh infection [9].However, laparoscopic approach in complicated groin hernia is technically demanding, likely due to difficulty in dealing with the irreducible/strangulated contents and reduced working space. [10].

In our study, most of the hernias (90.9%) were successfully reduced and the content of the hernia sac was mainly small bowel (50%) followed by omentum (36.4%). Most of the available literature have also shown successful reduction of the contents by different maneuvers, which may depend upon time of presentation and nature of hernia contents. In some cases there were evidence of automatic reduction of the contents post general anesthesia [6,[10], [11], [12]]. In contrast to our study, omentum was the primary content of the hernia sac in most of the previous studies [[13], [14], [15]].Combined laparoscopic approach allows sufficient time for gentle manipulation of contents during reduction along with careful inspection to detect any serosal tear or perforation of bowel, thus helping the surgeons to decide whether it needs resection [[5], [6], [7],^9^,^11^,^12^,^14^,^16^,^17^].Our study also had one case with bowel injury while manipulating the edematous bowel which was repaired primarily.

The decision to opt for laparoscopy or open approach in emergency setting depends upon the status of the patients as well as the expertise available in the center. Looking at developments over the years i.e. 2010 to 2019, laparoscopic approaches such as TAPP was used more often (21.9% in 2013 vs. 38% in 2019; p < 0.001), whereas the open approaches like Lichtenstein technique, Shouldice operation and “other techniques” were used less frequently. [11]In a study done by Yoon Yong Choi et al. TEP repair was suggested to be feasible in cases of complicated groin hernia [16].In 2009, Deebaet al*.,* carried out a systematic review on laparoscopic approach to incarcerated and strangulated inguinal hernia, which concluded that both TAPP and TEP were feasible and had comparable overall rate of complications, hernia recurrence and hospital stay as compared to those documented in open repair for strangulated hernia [12]. Hence, it can be assumed that the outcomes in our study cannot be attributed to the preference of one technique over other. The decision of choosing the type of procedure solely depended upon the treating surgeon's experience and expertise.

With the use of laparoscopic approach, the incidence of bowel resection rate can be minimized as we have plenty of time to observe the status of bowel. Studies done in the past have shown that unnecessary bowel resection can be minimized or avoided if laparoscopic approach is adopted in those scenarios [6,11]. Bowel resection rate in laparoscopic group was 1.7% versus 7.6% in the open group in a study comparing laparoscopic approach and open approach in complicated groin hernia [18].In our study, small bowel was the most of the hernia content (50%), which was successfully reduced and bowel resection was not required except in a single case where the content itself was gangrenous.

The complication rates for laparoscopic repair of uncomplicated groin hernia ranges from less than 3% to as high as 20%, ranging from minor (i.e. anesthesia related, wound infection, seroma, etc.) to major [i.e. vascular and nerve injury] [9,15,16,19].In our study, bleeding from inferior epigastric vessels and testicular vessels occurred in one patient (5.9%) each. Seroma and surgical site infection were seen in 2(11.8%) patients each. The complication rate in our study was even less as compared to laparoscopy repair in uncomplicated groin hernia which is really encouraging.

Mesh repair in complicated groin hernia decreases the chance of recurrence but its use is considered as a potential risk factor for infection. However, safety of mesh repair has been proven in various studies with fair results [6].Some studies suggest 85% of emergency groin hernia repair used mesh without substantial increase in the risks of postoperative complications [5,9,11,18]. In our study, mesh was kept in 90.9% of cases with no incidence of mesh infection or recurrence noted till date.

The mean total length of hospital stay in our study was 3.7 ± 3(range: 1 to 12) days which was slightly longer as compared to other studies [9,13,20]. Most of the patients included in our study were from remote rural areas with limited health service availability and hence the hospital stay was extended on patients' request.

As per our follow-up protocol for any case of hernia repair, the patients were followed up continually, including follow-up via telephonic conversation to assess about the persistence of pain and recurrence. The patients who underwent surgery during the early period were followed-up and data were entered and analyzed with a mean follow up period of 5.4 ± 2.2(range: 3 to 10) months, which was lesser as compared to most of the studies [9,13,15]. This shorter follow-up period is due to the limited time period of our study.

Only 2 patients (9.1%) in our study had moderate pain, comparable to previous studies. Systematic review of the literature conducted by Reinpold, 2017 showed chronic pain in 6% of cases [21]. In a study done by Yoon Young Choi et al., [2011], 3% cases had persistent postoperative pain [16].

During our study period, not a single case of recurrence was reported. Most of the studies shows recurrence rate ranging from 0.5% to 4.3% which was probably due to the long follow-up period.

Small sample size and short duration of post operative follow up are the limitations of our study. However, the investigators assessed for subjective symptoms of the participants till two years after the intervention. The examination at clinics were not feasible for later follow ups due to patient factors. Further, this study did not compare the outcomes of laparoscopy with other conventional techniques. Nonetheless, the improved outcomes from Laparoscopic hernia repair in complicated groin hernias shown for emergency setting in our study can serve as a basis for further prospective comparative studies.

## 5 Provenance and peer review

Not commissioned, externally peer reviewed.

## Funding source

This research did not receive any specific grant from funding agencies in the public, commercial, or not-for-profit sectors.

## Ethical approval

The study protocol was performed in relation to with the principles of the.

“Declaration of Helsinki” and after approval by the Institutional Review.

Committee and the Protocol Committee (Code No: IRC/1217/018).

## Sources of funding

This research did not receive any specific grant from funding agencies in the public, commercial, or not-for-profit sectors.

## Author contribution

Bhawani Khanal: Data analysis, Writing

Sunit Agrawal: Proof Reading

Robal lacoul: Study design, Data collection

Sashi shekhar Adhikari: Collection of materials

Abhijeet Kumar: Data analysis

Rakesh Kumar Gupta: Study design, Proof Reading.

## Trail registry number

1. Name of the registry: ClinicalTrials.gov.

2. Unique Identifying number or registration ID: NCT05107986.

3. Hyperlink to your specific registration (must be publicly accessible and will be checked): https://clinicaltrials.gov/ct2/show/NCT05107986?id=NCT05107986&draw=2&rank=1.

## Guarantor

Bhawani Khanal.

## International journal of surgery author disclosure form

The following additional information is required for submission. Please note that failure to respond to these questions/statements will mean your submission will be returned. If you have nothing to declare in any of these categories, then this should be stated.

## Declaration of competing interest

None.
